# Virtual Screening Approach to Identify High-Affinity Inhibitors of Serum and Glucocorticoid-Regulated Kinase 1 among Bioactive Natural Products: Combined Molecular Docking and Simulation Studies

**DOI:** 10.3390/molecules25040823

**Published:** 2020-02-13

**Authors:** Taj Mohammad, Shiza Siddiqui, Anas Shamsi, Mohamed F. Alajmi, Afzal Hussain, Asimul Islam, Faizan Ahmad, Md. Imtaiyaz Hassan

**Affiliations:** 1 Centre for Interdisciplinary Research in Basic Sciences, Jamia Millia Islamia, Jamia Nagar, New Delhi 110025, India; taj144796@st.jmi.ac.in (T.M.); anas.shamsi18@gmail.com (A.S.); aislam@jmi.ac.in (A.I.); fahmad@jmi.ac.in (F.A.); 2 Department of Biotechnology, Jamia Millia Islamia, Jamia Nagar, New Delhi 110025, India; shzsiddiqui26@gmail.com; 3 Department of Pharmacognosy College of Pharmacy, King Saud University, Riyadh 11451, Saudi Arabia; malajmii@ksu.edu.sa (M.F.A.); afzal.hussain.amu@gmail.com (A.H.)

**Keywords:** serum and glucocorticoid-regulated kinase 1, virtual screening, molecular docking, MD simulation, natural products, MM/PBSA

## Abstract

Serum and glucocorticoid-regulated kinase 1 (SGK1) is a serine/threonine kinase that works under acute transcriptional control by several stimuli, including serum and glucocorticoids. It plays a significant role in the cancer progression and metastasis, as it regulates inflammation, apoptosis, hormone release, neuro-excitability, and cell proliferation. SGK1 has recently been considered as a potential drug target for cancer, diabetes, and neurodegenerative diseases. In the present study, we have performed structure-based virtual high-throughput screening of natural compounds from the ZINC database to find potential inhibitors of SGK1. Initially, hits were selected based on their physicochemical, absorption, distribution, metabolism, excretion, and toxicity (ADMET), and other drug-like properties. Afterwards, PAINS filter, binding affinities estimation, and interaction analysis were performed to find safe and effective hits. We found four compounds bearing appreciable binding affinity and specificity towards the binding pocket of SGK1. The docking results were complemented by all-atom molecular dynamics simulation for 100 ns, followed by MM/PBSA, and principal component analysis to investigate the conformational changes, stability, and interaction mechanism of SGK1 in-complex with the selected compound ZINC00319000. Molecular dynamics simulation results suggested that the binding of ZINC00319000 stabilizes the SGK1 structure, and it leads to fewer conformational changes. In conclusion, the identified compound ZINC00319000 might be further exploited as a scaffold to develop promising inhibitors of SGK1 for the therapeutic management of associated diseases, including cancer.

## 1. Introduction

Serum/glucocorticoid-regulated kinase (SGK) is one of the serine/threonine-protein kinases found to be involved in the signaling pathway that regulates the sodium channel, cell survival, cell growth, proliferation, and cell migration [1]. It has three distinct isoforms, including serum and glucocorticoid-regulated kinase 1 (SGK1), SGK2, and SGK3, which are found in most vertebrates. SGK1 belongs to the AGC family of serine/threonine protein kinases, which is involved in the regulation of inflammation, apoptosis, enzymes transport, neuro-excitability, cell proliferation, and hormone release [2,3]. SGK1 has also been shown to activate the K^+^ channel, which further regulates several cellular processes, such as transport, cell proliferation, and neurotransmission [4]. SGK1 has significant sequence homology (45–60%) throughout its catalytic domain of other serine/threonine protein kinases, including protein kinase C family and Cyclic AMP-dependent protein kinases. It is constitutively expressed in a wide range of vertebrate tissues, with the highest levels in the thymus, ovary, and lung [5].

The *SGK1* gene is under the strict transcriptional control and its mRNA expression is rapidly induced in response to a variety of external stimuli viz., cell stress, and exposure to a variety of hormones, including glucocorticoid and mineralocorticoids [6]. Since SGK1 is regulated by a wide variety of signals, it has many functions and is reported to be involved in the regulation of several carriers and ion channels, including the epithelial sodium channel (EnaC), the renal outer medullary K^+^ channel (ROMK), the voltage-gated K^+^ and Na^+^ channel, the Na^+^/K^+^2Cl^−^ cotransporter (NKCC2), the glutamate transporters, etc. [7,8]. One of the mechanisms whereby SGK1 regulates channels is through the phosphorylation of Nedd4^−2^, a ubiquitin ligase that targets channels for degradation. Thus, it participates in the regulation of a wide variety of physiological processes, including epithelial transport, neuronal excitability, cell proliferation, and apoptosis [5].

Moreover, SGK1 regulates carrier and ion channel through phosphorylation by phosphoinositide-dependent protein kinase-1 (PDPK-1), a signaling intermediate downstream of PI3K, which in turn inhibits EnaC and promotes cancer cell proliferation [9]. The increased expression of SGK1 has been found in various tumors, including prostate cancer [10], colorectal cancer [11], and non-small cell lung cancer of the squamous subtype [12]. A study shows that RNA interference-mediated knockdown of SGK1 expression attenuates the androgen-mediated growth of the prostate cancer [10]. The overall observations suggest that SGK1 plays an important role in carcinogenesis and it can be considered as an attractive drug target for the development of anticancer therapeutics.

**Figure 1:**
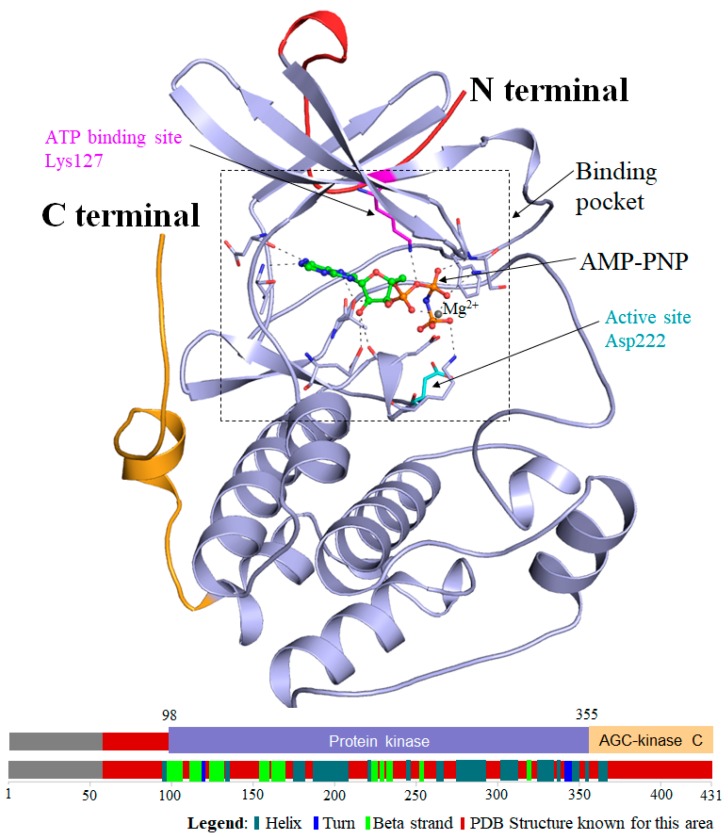
Structural organization of serum and glucocorticoid-regulated kinase 1 (SGK1). The overall structure of the SGK1 kinase domain in complex with co-crystallized AMP–PNP (adenosine 59(beta gamma-imido) triphosphate), and Mg^2+^. The N-terminal domain is in red, the C-terminal domain is in orange. AMP–PNP is shown in ball and stick model. Magnesium is represented by a grey sphere (upper). Schematic representation of the domain organization of SGK1 with secondary structural features (lower). The structure was drawn in PyMOL by using the atomic coordinates of SGK1 from the Protein Data Bank (PDB ID: 2R5T). The information about the domain organization was taken from UniProt (ID: O00141).

SGK1 is comprised of 431 amino acid residues with a molecular mass of 48,942 Da that has the quintessential bilobed kinase fold made up of an N-terminal β-strand domain and a C-terminal α-helical domain [5]. A hinge region that forms an important part of the catalytic site in SGK1 connects these two domains. The active site of SGK1 is Asp222, while Lys127 is the ATP binding site. SGK1 forms a dimer by two intermolecular disulfide bonds between Cys258 in the activation loop and Cys193 [5]. The SGK1 structure is similar to the common protein kinase fold, but the conformation around the active site is distinctive when compared to other protein kinases [5]. Figure 1 illustrates the structural organization of SGK1. Since the differences in SGK1 from other kinases occur around the ATP-binding site, this structure can provide valuable insight into the designing and development of selective and highly potent competitive inhibitors of SGK1.

The commercially available SGK1 inhibitors i.e., EMD638683 [13,14] and GSK650394 [10], are being evaluated under clinical trials. EMD638683 (*N’*-[2-(3,5-Difluorophenyl)-2-hydroxyacetyl]-2-ethyl-4-hydroxy-3-methylbenzohydrazide) is a potent SGK1 inhibitor, with an IC_50_ value of 3 μM. EMD638683 is also found to inhibit cAMP-dependent protein kinase [1], mitogen- and stress-activated protein kinase 1 (MSK1), protein kinase C-related kinase 2 (PKR2), and the SGK isoforms SGK2 and SGK3 [13]. GSK650394 (2-Cyclopentyl-4-(5-phenyl-1H-pyrrolo[2,3-b]pyridin-3-yl)-benzoic acid) is another inhibitor of SGK1, with an IC_50_ value of 62 nM in the scintillation proximity assay [10]. It shows > 30-fold selectivity for SGK1 over Akt and other related kinases [10]. It has been found that GSK650394 can inhibit the androgen-stimulated growth of human prostate carcinoma LNCaP cells with an IC_50_ value of ~1 μM [10].

There is a great scope to develop safer and highly specific SGK1 inhibitors with improved pharmacological properties while using computational methods [15,16,17]. Structure-based drug design has become a useful and essential part of the drug discovery and possibly the most relevant approach to discover bioactive leads exhibiting high specificity and effectiveness [18,19]. Virtual high-throughput screening (vHTS) is a widely adopted approach in drug discovery used for the identification of new leads [20,21]. It is a computational screening method that is used to identify druggable candidates from the collection of certain chemical libraries via searching the ligands binding to a target protein with high affinity. This technique is cost-effective and reliable, being used in the identification of potential leads and it avoids undesirable compounds that would otherwise result in expensive and time-consuming experimentation [22].

Recent advances in structural biology have revolutionized the screening of natural products in the discovery of novel, safe, and innovative drugs [23,24,25]. Natural compounds have been recognized as an incredible source of lead for drug discovery, because they possess a wide range of chemical structures, biochemical specificity, and other molecular features [26,27]. In the present study, we have taken 90,000 natural compounds from the ZINC database and filtered by applying Lipinski’s rule of five. These compounds were further filtered for their ADMET properties, carcinogenicity, and PAINS patterns. Subsequently, we have performed structure-based vHTS of filtered compounds against SGK1 and analyzed their binding patterns in detail. We further assess the structural flexibility and dynamic stability of SGK1 in the presence of the selected compound by utilizing molecular dynamics (MD) simulation, Molecular mechanics Poisson–Boltzmann surface area (MM/PBSA), and principal component analysis. We performed all-atom MD simulations for 100 ns on apo and ligand-bound state of SGK1 to describe their interaction and conformational changes in SGK1 for the systematic evaluation of their dynamic behavior under the explicit solvent condition.

## 2. Results and Discussion

### 2.1. Filtration of Natural Products

The physicochemical properties of all the compounds that were present in the ZINC library were calculated through the SwissADME and Discovery Studio, where we filtered out 32,902 natural compounds passed in the Lipinski’s rule of five (Molecular weight ≤ 500 Da, logP ≤ 5, number of hydrogen bond donor ≤ 5 and hydrogen bond acceptor ≤ 10) and having good bioavailability score and zero PAINS pattern. Table 1 shows the physicochemical properties of the finally selected four compounds. The identified four compounds show better physicochemical properties when compared to the known SGK1 inhibitor GSK650394. However, GSK650394 violates the Rule of five, as it possesses the logP of 6.25, making it a poorly soluble compound in polar solvents.

The compounds were further screened to predict the Absorption, Distribution, Metabolism, Elimination, and Toxicity (ADMET) properties, along with their carcinogenicity to identify more bioavailable and non-carcinogenic compounds. ADMET properties of the finally selected four compounds predicted through the pkCSM webserver [28] showing parameters assessed were within the range of draggability, as shown in Table 2. In the comparison of the ADMET properties of identified compounds with the known SGK1 inhibitors, compound ZINC00319000 showed better ADMET properties than EMD638683 and GSK650394. EMD638683 showed similar ADMET properties, except lower GI absorption than the identified compounds. While GSK650394 is found to be poorly soluble in water and showing toxicity, as predicted (Table 2).

### 2.2. vHTS

The vHTS results in the identification of several compounds having appreciable docking scores with SGK1. We have selected the top 30 hits out of 32,902 natural compounds showing an appreciable binding affinity with SGK1. The identified hits were showing binding affinity within the range of −10.9 to −11.8 kcal/mol towards SGK1 (Table 3). Finally selected four compounds ZINC00319000, ZINC02106991, ZINC02115387, and ZINC02121074 are binding to SGK1 with an affinity of −10.9 kcal/mol, −10.9 kcal/mol, −10.9 kcal/mol, and −11.0 kcal/mol, respectively. All four compounds showed higher affinity towards SGK1 as compared with known SGK1 inhibitors, EMD638683 and GSK650394 (Table 3).

**Figure 2:**
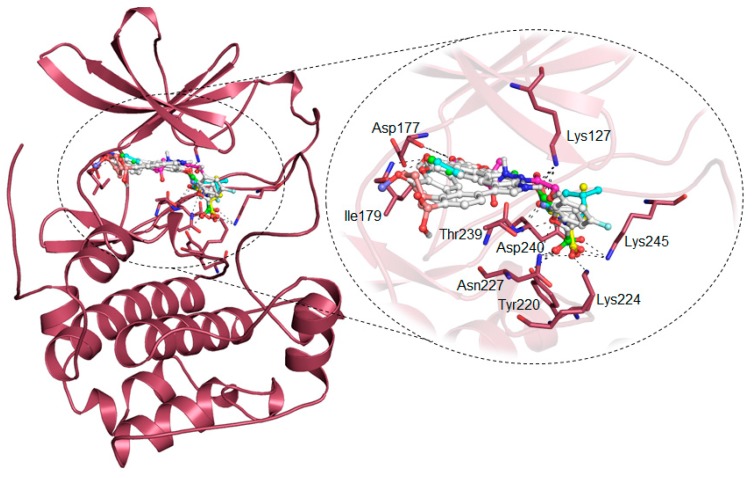
Cartoon representation of SGK1 in-complex with compound ZINC00319000 (green element), ZINC02106991 (cyan element), ZINC02115387 (yellow element), ZINC02121074 (blue element), EMD638683 (magenta element), and GSK650394 (red element).

**Figure 3:**
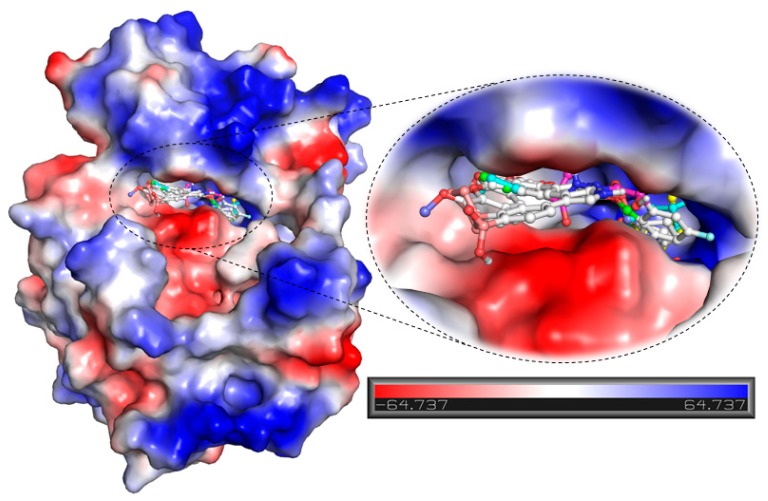
Potential surface representation of SGK1 in-complex with compound ZINC00319000 (green element), ZINC02106991 (cyan element), ZINC02115387 (yellow element), ZINC02121074 (blue element), EMD638683 (magenta element), and GSK650394 (red element).

**Figure 4:**
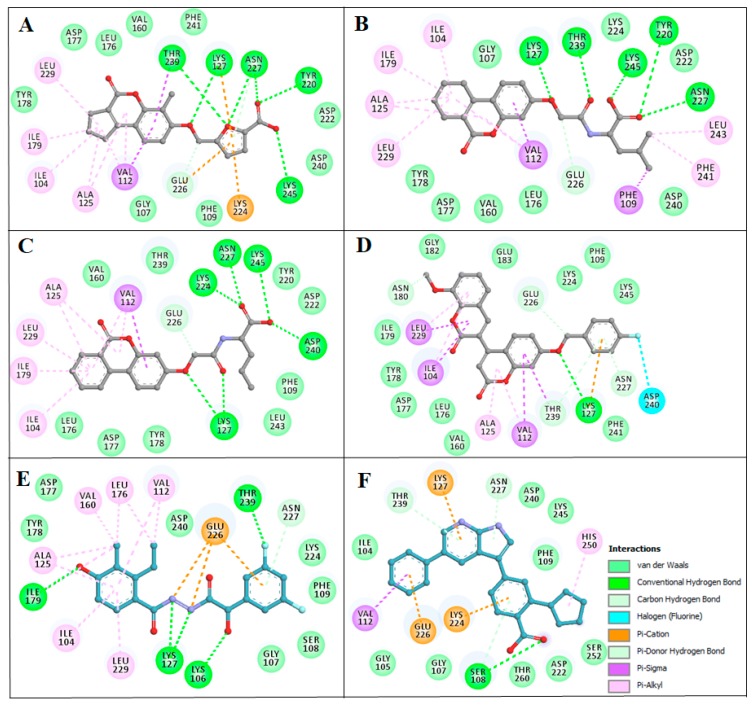
Two-dimensional (2D) structural representation of SGK1 residues interacting to the compound (**A**) ZINC00319000, (**B**) ZINC02106991, (**C**) ZINC02115387, (**D**) ZINC02121074, (**E**) EMD638683, and (**F**) GSK650394.

Further, detailed interaction analysis of all the possible docked conformers (a total of 270) of the top 30 hits was done based on their specific interaction towards the SGK1 binding pocket. Here, we identified four compounds that have commonly interacted with a set of functionally active residues of SGK1, where co-crystallized ligand Phosphoaminophosphonic Acid-Adenylate Ester is binding [5]. It has been observed that residues of the kinase domain of SGK1, such as Lys127, Asn227, Lys224, Ile179, Asp177, Glu226, Phe109, Ser108, Thr239, Tyr220, and Lys245 offer a significant number of interactions to the selected compounds. These observations suggest that our selected compounds also mimick the same orientation of the co-crystallized ligand. The identified compounds and EMD638683 and GSK650394 showed a similar pattern of binding. Figure 2, Figure 3 and Figure 4 present a detailed interaction of the finally selected four compounds and their binding pattern with SGK1.

Compounds that were docked to the binding pocket of SGK1 were checked for their interaction with the functionally important residues of the protein. The ATP binding-site residue Lys127 and the active site residue Asp222 located on the main catalytic center of the protein are responsible for the functional activity of SGK1 [5]. It is evident from Figure 2 that all the selected compounds significantly interact with Lys127 and Asp222. All of the compounds are showing common interactions as EMD638683 and GSK650394. A significant number of specific interactions formed between these functionally important residues and the identified compounds, suggesting a strong binding affinity and their further implications as ATP/substrate-competitive inhibitors of SGK1. Overall, the physicochemical, predicted ADMET properties, and interaction analysis in comparison of the known SGK1 inhibitors (EMD638683 and GSK650394) suggest that the identified four compounds (ZINC00319000, ZINC02106991, ZINC02115387, and ZINC02121074) can be further evaluated as potent scaffold in development of highly selective SGK1 inhibitors with improved pharmacological properties (Table 4).

### 2.3. Biological Activity Predictions

The prediction of biological activity while using the PASS webserver resulted in similar kinds of biological activities of the selected compounds. In this analysis, the reference compounds, EMD638683 and GSK650394, showed SGK inhibitory potential, validating the results found in the literature [10,13]. The molecules ZINC00319000, ZINC02106991, ZINC02115387, and ZINC02121074 have shown satisfactory predictions for anticancer, anti-inflammatory, antiarthritic, and kinase inhibitory potential, with Pa ranging from 0.375 to 0.787 when Pa > Pi. Table 4 shows the predicted activities of the selected compounds with higher Pa value.

### 2.4. Structure-Activity Relationship of the Compounds

The structure–activity relationship is the connection between the molecular structure of a compound and its biological activity, which has been utilized in structure-based drug designing to develop effective small molecules against a biological target [29]. There are two known SGK inhibitors, namely GSK650394 and EMD638683, which have effective characteristics to inhibit the activity of SGK1. The compounds ZINC00319000, ZINC2106991, ZINC02115387, and ZINC02121074 show their molecular structures very similar to the known SGK1 inhibitors, for example, GSK650394, which has a similar structure to the compounds investigated here. Importantly, GSK650394 is relatively nontoxic in nature and has been found to inhibit SGK1 with an IC_50_ value of 62 nM in the scintillation proximity assay [10]. The promising molecule, ZINC00319000, has a close relationship between its structure with the known SGK1 inhibitor GSK650394 (Table 5). It can be hypothesized from the structure-activity relationship that the identified compound ZINC00319000 might have high potential to inhibit SGK1 activity and can be further improved to develop highly selective inhibitors of SGK1.

Pharmacophore mapping has become one of the major components of the drug discovery process. A number of ligand-based and structure-based methods have been developed for pharmacophore modeling and being extensively utilized in structure-based virtual-screening and lead optimization [30]. The pharmacophoric features of the selected compounds and known SGK1 inhibitors showed a high similarity between their molecular properties, as shown in Table 6. The pharmacophore features, such as Donors, Acceptors, Aromatic, and Hydrophobic groups, are actively participating in various interactions with SGK1, as shown in Figure 4.

### 2.5. MD Simulations

One of the identified compounds, ZINC00319000, in complex with SGK1 along with the SGK1 in apo-state was subjected to MD simulation for 100 ns. The average potential energy of SGK1 apo and SGK1-ZINC00319000 was calculated to ascertain the equilibration and stability of the systems. The average potential energy for SGK1 apo and SGK1-ZINC00319000 complex was found to be −974,994 kJ/mol and −974,946 kJ/mol, respectively. Several systematic and energetic parameters, including volume, density, kinetic energy, enthalpy, and total energy, were also estimated after the simulation (Table 7).

### 2.6. Structural Deviations and Compactness

**Figure 5:**
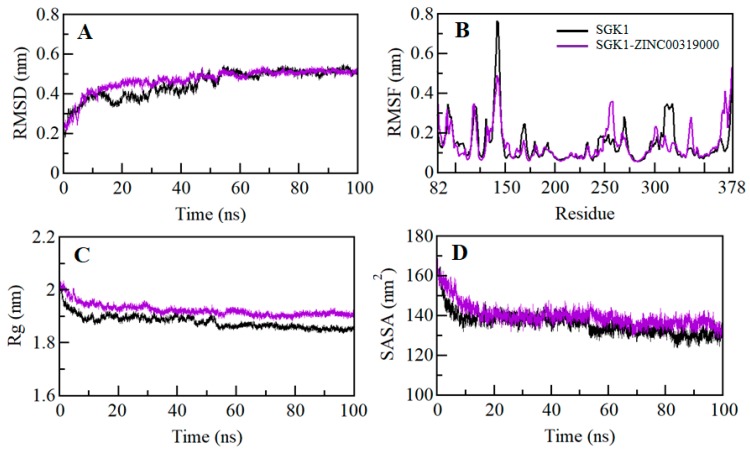
Structural dynamics of SGK1 as a function of time. (**A**) Root-mean-square deviation (RMSD) plot of SGK1 before and after binding of ZINC00319000. (**B**) Residual fluctuations plot of SGK1 before and after ZINC00319000 binding. (**C**) Time evolution of the radius of gyration. (**D**) SASA plot of SGK1 as a function of time. The values were obtained from the 100 ns molecular dynamic (MD) simulation time scale. Black and violet represent values obtained for SGK1 apo and SGK1-ZINC00319000 complex, respectively.

A small molecule can induce large conformational deviations to a protein, after binding. Root-mean-square deviation (RMSD) is one of the fundamental properties for exploring structural changes and the dynamic behavior of protein structure [31]. The average RMSD for SGK1 apo and SGK1-ZINC00319000 complex were calculated and found to be 0.45 nm and 0.46 nm, respectively (Table 7). The RMSD plot shows that the SGK1 is getting stabilized after the binding of compound ZINC00319000 as compared with the free SGK1. The binding of ZINC00319000 leads to fewer structural deviations in SGK1 from its native conformation and stabilized throughout the simulation trajectory (Figure 5A). However, a little increment in RMSD of SGK1 can be seen from 15 to 50 ns after compound binding, which might be due to the initial orientation of ZINC00319000 in the binding pocket of SGK1. The RMSD of SGK1 in-presence of ZINC00319000 is showing equilibration, with no switching throughout the trajectory, which suggests strong stability of the SGK1-ZINC00319000 complex (Figure 5A).

The average fluctuation of each residue was calculated as root-mean-square fluctuation (RMSF) to investigate the residual vibrations in SGK1 before and after the binding of the compound ZINC00319000 (Figure 5B). We noticed that random residual fluctuations are present in SGK1 at different regions, spanning from N- to C-terminal. These fluctuations were plotted after the simulation for each residue in the backbone of SGK1 before and after ZINC00319000 binding. The residual fluctuations found to be minimized in the case of the SGK1-ZINC00319000 complex. The analysis of the RMSF plot suggested that the residual fluctuations are showing minimal changes in the region where ZINC00319000 is binding. However, fewer increased fluctuations are also seen in SGK1 after ZINC00319000 binding possibly due to its conformation’s adjustment in the binding pocket of the protein.

The radius of gyration (*R_g_*) is directly associated with the tertiary structure and overall conformational state that has been utilized to understand the compactness and folding behavior of a protein. We assessed the stability of SGK1 and SGK1-ZINC00319000 complex by computing the *R_g_* of both systems. The average *R_g_* for SGK1 apo and SGK1-ZINC00319000 complex was calculated as 1.88 nm and 1.92 nm, respectively. The *R_g_* plot shows a minor increment in *R_g_* values up to 0.04 nm, while compound ZINC00319000 binds to SGK1, which is possibly due to its packing. No structural switching was observed in SGK1 in the presence of ZINC00319000, and it attained stable *R_g_* equilibrium, thus suggesting complex stability throughout the simulation trajectory (Figure 5C).

The Solvent-accessible surface area is the interface between a protein and its surrounding solvent due to its electrostatic and surface properties [32]. The solvent on the surface of a system might behave differently in different conditions and it can be used to investigate the conformational dynamics in a protein under solvent conditions. We have computed the SASA of SGK1 apo and SGK1-ZINC00319000 complex to investigate their conformational behavior during the simulation. The average SASA for SGK1 in apo and SGK1-ZINC00319000 complex was found to be 135.94 nm^2^, and 139.74 nm^2^, respectively. A little increment in SASA was observed, possibly due to increased surface area of SGK1 in the presence of ZINC00319000, where some inner residues might be exposed to the surface (Figure 5D). The SASA attained stable equilibrium without switching throughout the simulation, thus suggesting the structural stability of SGK1 in the presence of compound ZINC00319000.

### 2.7. Dynamics of SGK1 Interactions: Hydrogen Bonds Analysis

**Figure 6:**
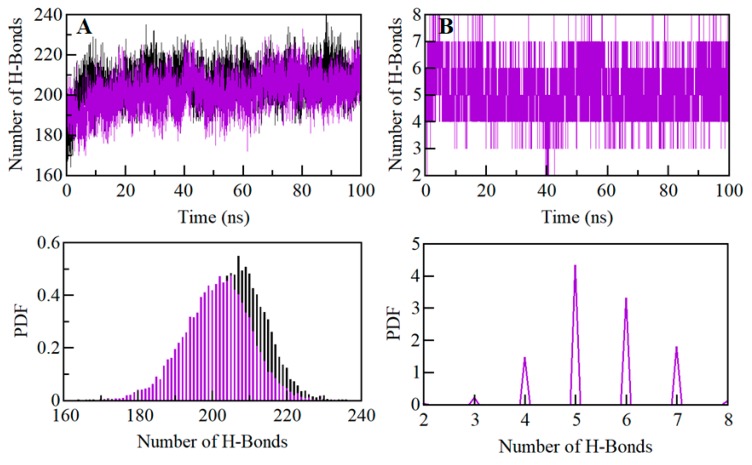
Time evolution and stability of hydrogen bonds formed (**A**) Intramolecular within SGK1, and (**B**) Intermolecular between compound ZINC00319000 and SGK1 (lower panel shows the probability of distribution of hydrogen bonding as PDF).

Intramolecular hydrogen bonds within a protein molecule play a fundamental role in defining the stability of the three-dimensional structure of a protein [33]. Hydrogen bonds analysis can also be utilized to investigate the stability of the protein-ligand complex in the evaluation of molecular recognition, directionality, and specificity of interactions [33]. We have computed the dynamics of intramolecular hydrogen bonds paired within 0.35 nm to validate the stability of SGK1 apo and SGK1-ZINC00319000 docked complex. The average number of intramolecular hydrogen bonds in SGK1 before and after ZINC00319000 binding was found to be 206 and 205, respectively (Figure 6A). A little decrement in hydrogen bonding within SGK1 itself is might be due to the occupation of some intramolecular space of binding pocket by the compound ZINC00319000. We also calculated the probability distribution function (PDF) of hydrogen bond dynamics, where the analysis indicates that the complex of SGK1-ZINC00319000 is quite stable with minimal change (Figure 6A, lower panel). We also analyzed the dynamics of intermolecular hydrogen bonds between ZINC00319000 and SGK1 paired within 0.35 nm to investigate the complex stability. We found an average of five intermolecular hydrogen bonds shared by the compound ZINC00319000 to SGK1, which were formed throughout the simulation (Figure 6B). The analysis revealed that compound ZINC00319000 binds in the active pocket of SGK1 with 7–8 hydrogen bonds with fluctuation and 5–6 hydrogen bonds with higher stability, which also supports our docking finding. The PDF of intermolecular bonding shows that five hydrogen bonds formed between compound ZINC00319000 and SGK1, with higher stability and distribution, throughout the simulation trajectory (Figure 6B, lower panel).

### 2.8. Secondary Structure Dynamics of SGK1

**Figure 7:**
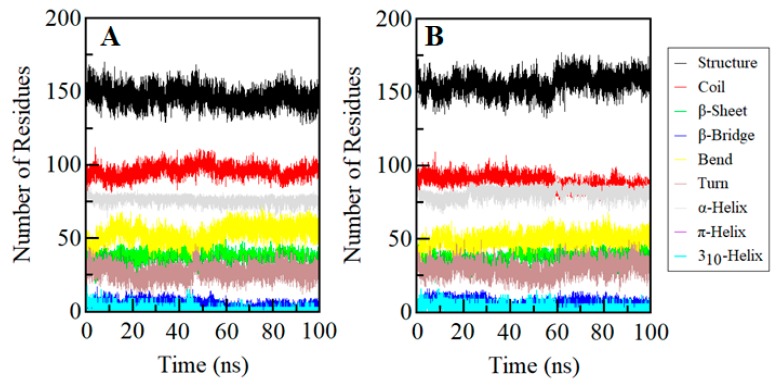
Secondary structure content in (**A**) Free SGK1 and (**B**) SGK1 upon ZINC00319000 binding. Structure = α-helix + β-sheet + β-bridge + Turn.

Conformational changes in a protein structure are the result of the varying degrees of dynamic residual secondary structure. Getting insight into the changes in the secondary structure content of a protein can be utilized to understand the conformational behavior and folding mechanism of their polypeptide chain. We have analyzed the dynamics of the secondary structure content of SGK1 to investigate its stability before and after ZINC00319000 binding. The secondary structure elements viz., α-helix, β-sheet, and turn of SGK1 were broken into individual residues at each time step, and the average number of residues forming secondary structure was plotted as a function of time. The analysis shows that the structural elements of SGK1 in the free state remain almost constant and equilibrated during the simulation (Figure 7). Whereas, a little increment in the secondary structure content of SGK1 can be seen while in-complex with ZINC00319000 (Figure 7B). This increment in average secondary structure content is mainly due to the conversion of coils into α-helix (Table 8). Overall, no major changes were seen in the secondary structure content of SGK1 upon ZINC00319000 binding, which suggests the strong stability of the complex.

### 2.9. Principal Component and Free Energy Landscape Analysis

**Figure 8:**
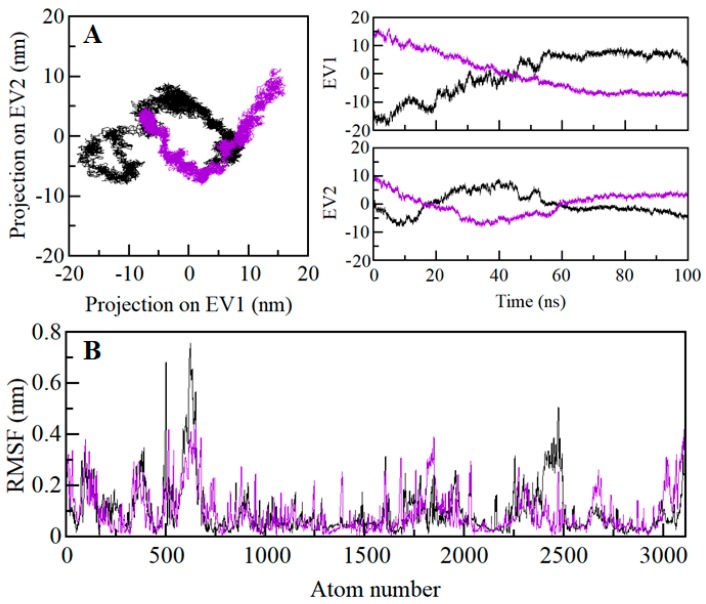
Principal component analysis. (**A**) 2D projections of trajectories on eigenvectors showed different projections of SGK1 (left panel). The projections of trajectories on eigenvectors with respect to time (right panel). (**B**) Atomic fluctuations of SGK1 on eigenvector 1. Black and violet represent the values obtained for free SGK1 and SGK1-ZINC00319000 complex, respectively.

Proteins do the collective motion in their atoms to perform specific functions. We have performed principal component analysis (PCA) to investigate the conformational sampling of SGK1 and SGK1-ZINC00319000 complex via examining their collective motions while using the essential dynamics approach [34]. The dynamics of a protein can be illustrated through their phase space behavior. Eigenvalues were extracted through the covariance matrix and the principal components (PCs) while using the *gmx anaeig* and *gmx covar* tools to investigate the principal motion directions in essential subspace. The eigenvalues were extracted corresponding to each eigenvector (EV) that inductive for the direction of motion in essential phase space. The conformational sampling of SGK1-apo and SGK1-ZINC00319000 complex in the essential subspace is shown in Figure 8 which illustrates the tertiary conformations along with the EV1 and EV2 projected by the C^α^ atom. Here, we found that the SGK1-ZINC00319000 complex occupied a notable different conformational subspace when compared to the SGK1 in the free state. From Figure 8A, it can be observed that the overall flexibility of the SGK1-ZINC00319000 complex was increased at PC1 with a significant overlapping of stable clusters with phase space of SGK1-apo. The PCA analysis indicates that SGK1 in-complex with compound ZINC00319000 is quite stable with increased dynamics of its structural conformation.

**Figure 9:**
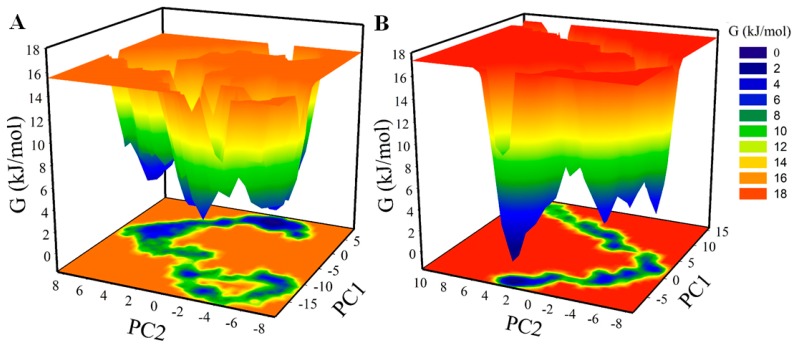
The Gibbs energy landscape obtained during 100 ns MD simulation for (**A**) free SGK1 and (**B**) SGK1-ZINC00319000 complex.

The Gibbs free energy landscapes (FELs) were analyzed while using the first two EVs to further investigate the conformational behavior of SGK1. Figure 9 displays the FELs of SGK1 and the complex system of SGK1 in-presence of ZINC0031900. The deeper blue in the plots is indicating the different conformational states having lower energy. SGK1 only shows a single global minimum confined within a single local basin. However, SGK1 in the presence of ZINC0031900 acquired different conformational motion with noticeable change and did not progress to multiple stable global minima. The analysis shows that the presence of ZINC0031900 affects the size and the position of the sampled essential space of SGK1 with a stable single global minimum (Figure 9B).

### 2.10. MPBSA Analysis

The quantitative estimation of the binding free energy of compound ZINC00319000 to SGK1 was carried out while using the MMPBSA method. A stable short region of 90 to 100 ns from the simulated trajectory of the docked complex was extracted for the *g_mmpbsa* calculations while using polar and apolar solvation parameters. This analysis aims to estimate the energies that are associated with the binding of ZINC00319000 to SGK1 during the MD simulation. The binding energy for the SGK1-ZINC00319000 complex was estimated to be −132.49 ± 13.82 kJ/mol. The MMPBSA analysis suggests that the compound ZINC00319000 binds to SGK1 with an appreciable binding affinity and result in the formation of a stable complex.

## 3. Materials and Methods

### 3.1. Computational Resources

Various bioinformatics software, such as MGL Tools [35], AutoDock Vina [36], Discovery Studio [37], and GROMACS 5.1.2 [38], were used for vHTS and MD simulation studies. Several online and standalone resources, like National Center for Biotechnology Information [39], RCSB Protein Data Bank (PDB) [40], ZINC database [41], SwissADME [42], CarcinoPred-EL [43], VMD [44], SPDBV [45], and QtGrace [46] were used in retrieval, evaluation, and analysis of the data. The crystal structure of human SGK1 was retrieved from the PDB (PDB ID: 2R5T, Resolution: 1.9 Å) and refined further while using SPDBV and MGL tools. The library of natural products containing ~90,000 compounds was obtained from the ZINC database in processed format. The pharmacophore features of the selected compounds were generated through the PharmaGist Webserver (https://bioinfo3d.cs.tau.ac.il/PharmaGist/). Detailed information on pharmacophore modeling and structure-based virtual screening have been described elsewhere [47,48,49].

### 3.2. Filtration of Natural Products

All the compounds that were present in the ZINC library were filtered on the basis of their physicochemical properties through the SwissADME and Discovery Studio. Initially, we selected compounds based on their physicochemical properties satisfying the Lipinski’s rule of five [50]. We applied the PAINS filter to avoid compounds with PAINS patterns that can have a higher tendency to bind with multiple targets. We further screened the compounds for their carcinogenic patterns and ADMET properties. Compounds having well ADMET properties and non-carcinogenic patterns were only selected for structure-based vHTS.

### 3.3. Structure-Based vHTS

Three-dimensional coordinates of human SGK1 were taken from the PDB (PDB ID: 2R5T) and heteroatoms, including co-crystallized ligand AMP-PNP and water molecules, were removed. The parent structure from the PDB has four mutations and some missing residue from aa Lys136 to Asn148. Therefore, the structure was remodeled while using the wildtype sequence of SGK1 (UniProt identifier: O00141-1) through MODELLER 9.21 to fill the break by taking the original structure as a template (PDB ID: 2R5T) and further refined while using the SPDBV and the MGL tools. Hydrogens were added to the polar groups in the protein, along with the Kollman charges. The docking was performed while using AutoDock Vina, where the screening was structurally blind for all of the compounds with a grid size of 65, 57 and 104 Å, centralized at 29.123, 34.392, and 72.13 for X, Y, and Z coordinates, respectively. The grid spacing was set to 1.00 Å with the exhaustiveness of 8. The filtered library of natural compounds was used to perform structure-based vHTS to screen compounds based on their binding affinities towards SGK1. The docking results were screened for higher binding affinity, and all possible docked conformations were then generated for each compound, which was further analyzed while using PyMOL and Discovery Studio for their possible interaction towards SGK1. From the interaction analysis, the only compounds selected that were specifically interacting with the binding-site and active-site residues of SGK1 [51,52,53]. Here, we found four potential compounds interacting with the binding pocket of SGK1.

### 3.4. Biological Activity Predictions of the Compounds

The predictions of biological activities of the selected compounds were performed while using the PASS webserver http://www.pharmaexpert.ru/passonline [54]. The PASS analysis allows us to discover the effects of a compound based entirely on the molecular formula while using MNA (multilevel neighbors of atoms) descriptors, which suggested that the biological activity is the function of its chemical structure [49,55].

### 3.5. MD Simulations

All-atom MD simulation was performed on SGK1 before and after the binding of one of the identified compounds i.e., ZINC00319000 for 100 ns at 300 K at the molecular mechanics level while using GROMOS 54A7 force-field in GROMACS 5.1.2. The topology parameters for compound ZINC00319000 were generated through an external web-resource the PRODRG server and then merged into the protein topology that was generated through the GROMACS to make SGK1-ZINC00319000 complex system. SGK1 and SGK1-ZINC00319000 systems were both solvated in a cubic box with the Simple Point Charge (spc216) water model to simulate in aqueous surroundings as described [56,57,58]. Energy minimization was completed to remove the possible steric clashes in the systems while using 1500 steps of the steepest descent method for 1000 ps. The temperature of both systems was subsequently raised from 0 to 300 K during the equilibration period of 100 ps at constant volume under periodic boundary conditions with a stable environment of 1-bar pressure. Final MD run was performed for 100,000 ps for both systems, and resulting trajectories were analyzed while using the inbuilt utilities of GROMACS and visualized in VMD and QtGrace.

### 3.6. MMPBSA Calculations

Molecular mechanics/Poisson-Boltzmann surface area (MMPBSA) is one of the most widely used approaches for estimating the binding free energy of a protein-ligand complex [59]. A short MD trajectory of 10 ns (from 90 to 100 ns) was extracted from the stable region of the SGK1-ZINC00319000 complex for MMPBSA calculations [60]. The binding energy components were calculated while using the MMPBSA approach of the *g_mmpbsa* package [61]. The *g_mmpbsa* tool uses the following equation to calculate the binding energy of the protein-ligand complex-

Δ*G*_Binding_ = *G*_Complex_ − (*G*_Protein_ + *G*_Ligand_)

where, *G*_Complex_ signifies the total free energy of the binding complex, and *G*_Protein_ and *G*_Ligand_ are the measure of total free energies of SGK1 and compound ZINC00319000, respectively.

## 4. Conclusions

The world is facing multiple health challenges with the emergence of several complex diseases, such as cancer and neurodegenerative disorders. This work aims to contribute to the therapeutic management of cancer by using natural products in the development of novel anticancer drugs. SGK1 is a positive regulator of cancer progression, proliferation, and migration, and, thus, can be utilized as an attractive drug target for the development of anticancer therapeutics. In the present study, an *in-silico* analysis using structure-based vHTS of the ZINC database of natural products against SGK1 was carried out to identify its potent inhibitors, which can further be used in the development of potential drugs against cancer. The selected four natural compounds (ZINC00319000, ZINC2106991, ZINC02115387, and ZINC02121074) were filtered by assessing their physicochemical and drug-like properties, showing appreciable binding affinities towards the binding site of SGK1. The PASS analysis has predicted satisfactory biological activities (anticancer, anti-inflammatory, antiarthritic, and kinase inhibitory potential) of the promising molecules investigated in this study. Interaction analysis showed that selected compounds bind to a common set of amino acid residues of the SGK1 binding pocket. The structure-activity relationship showed a close relationship between the chemical structures of the compound ZINC00319000 and the known SGK1 inhibitor GSK650394. MD simulation studies confirm that compound ZINC00319000 efficiently binds to SGK1 and forms a stable complex with minimal structural changes. The identified compound ZINC00319000 can be further validated in *in-vitro* studies and can subsequently be utilized for the development of anticancer therapeutics. Our approach will be proved useful in the designing of drugs while using natural leads as potent inhibitors of SGK1 for the therapeutic management of cancer and other associated diseases.

## Figures and Tables

**Table 1:** The physicochemical properties of four potentially selective compounds against SGK1.

Compound ID.	Molecular Weight	No. of Rotatable Bonds	No. of H-Bond Donor	No. of H-Bond Acceptor	logP	Lipinski Violation
**ZINC00319000**	340.33	4	1	5	3.10	0
**ZINC02106991**	387.43	7	2	5	3.48	0
**ZINC02115387**	369.37	7	2	5	3.23	0
**ZINC02121074**	444.41	5	0	6	4.62	0
**EMD638683**	364.34	4	4	6	2.03	0
**GSK650394**	382.46	4	2	3	6.25	1

**Table 2:** Absorption, Distribution, Metabolism, Elimination, and Toxicity (ADMET) properties of the selected compounds.

Compound ID	Absorption		Distribution	Metabolism	Excretion	Toxicity
	GI Absorption (%)	Water Solubility	BBB/CNS Permeation	CYP2D6 Inh/Subs	OCT2 Substrate	AMES Toxicity
**ZINC00319000**	95.20	Soluble	No	No	No	No
**ZINC02106991**	59.18	Soluble	No	No	No	No
**ZINC02115387**	60.62	Soluble	No	No	No	No
**ZINC02121074**	99.84	Soluble	No	No	No	No
**EMD638683**	60.54	Soluble	No	No	No	No
**GSK650394**	98.96	Poor	No	No	No	Yes

**Table 3:** List of the top 30 hits selected based on their binding affinity towards SGK1.

S. No.	Compound ID	Affinity (kcal/mol)
**1.**	ZINC02092709	−11.8
**2.**	ZINC02095133	−11.7
**3.**	ZINC02119552	−11.7
**4.**	ZINC02116493	−11.4
**5.**	ZINC02129029	−11.4
**6.**	ZINC02127993	−11.3
**7.**	ZINC02131906	−11.2
**8.**	ZINC02092851	−11.1
**9.**	ZINC02114667	−11.1
**10.**	ZINC02127995	−11.1
**11.**	ZINC02136713	−11.1
**12.**	ZINC00848446	−11.0
**13.**	ZINC01105783	−11.0
**14.**	ZINC02114669	−11.0
**15.**	ZINC02117876	−11.0
**16.**	ZINC02119756	−11.0
**17.**	ZINC02120975	−11.0
**18.**	ZINC02121074	−11.0
**19.**	ZINC02130599	−11.0
**20.**	ZINC02137640	−11.0
**21.**	ZINC02149843	−11.0
**22.**	ZINC00319000	−10.9
**23.**	ZINC02096281	−10.9
**24.**	ZINC02106991	−10.9
**25.**	ZINC02112637	−10.9
**26.**	ZINC02114126	−10.9
**27.**	ZINC02115387	−10.9
**28.**	ZINC02117188	−10.9
**29.**	ZINC02117586	−10.9
**30.**	ZINC02120692	−10.9
**31.**	EMD638683	−8.6
**32.**	GSK650394	−9.1

**Table 4:** Biological activity prediction of the selected compounds along with the known SGK1 inhibitors.

Compound ID.	Pa	Pi	Biological Activity
**ZINC00319000**	0.515	0.053	Anti-inflammatory
**ZINC02106991**	0.787	0.009	Antiarthritic
**ZINC02115387**	0.751	0.012	Antiarthritic
**ZINC02121074**	0.375	0.080	Kinase inhibitor
**EMD638683**	0.376	0.004	SGK inhibitor
**GSK650394**	0.740	0.002	SGK1 inhibitor

Pa = probability to be active; Pi = probability to be inactive.

**Table 5:** List of the identified compounds along with known SGK1 inhibitors (EMD638683 and GSK650394) and their chemical structures.

Compound ID	Chemical Name	Molecular Formula	Structure
**ZINC00319000**	5-[(methyl-oxo-BLAHyl) oxymethyl]furan-2-carboxylic	C_19_H_16_O_6_	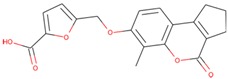
**ZINC2106991**	(2*S*)-2-[[2-[(6-keto-7,8,9,10 tetrahydrobenzo[c]isochromen-3-yl)oxy]acetyl]amino]-4-methyl-valerate	C_21_H_25_NO_6_	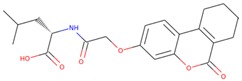
**ZINC02115387**	(2*S*)-2-[[2-(6-ketobenzo[c]isochromen-3-yl)oxyacetyl]amino]valerate	C_20_H_19_NO_6_	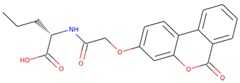
**ZINC02121074**	3-[7-(4-fluorobenzyl)oxy-2-keto-chromen-4-yl]-8-methoxy-coumarin	C_26_H_17_FO_6_	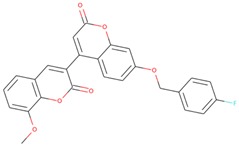
**EMD638683**	*N’*-[2-(3,5-Difluorophenyl)-2-hydroxyacetyl]-2-ethyl-4-hydroxy-3-methylbenzohydrazide	C_18_H_18_F_2_N_2_O_4_	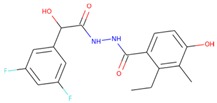
**GSK650394**	2-cyclopentyl-4-(5-phenyl-1*H*-pyrrolo[2,3-b]pyridin-3-yl)benzoic acid	C_25_H_22_N_2_O_2_	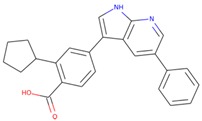

**Table 6:** Spatial pharmacophore features of the selected compounds along with known SGK1 inhibitors (EMD638683 and GSK650394).

Molecule	No. of Atoms	Spatial Features	Aromatic	Hydrophobic	Donors	Acceptors	Negative Group	Positive Group
**ZINC00319000**	40	15	3	5	2	5	1	0
**ZINC2106991**	52	19	2	9	1	5	1	0
**ZINC02115387**	45	13	2	3	2	5	1	0
**ZINC02121074**	50	12	4	2	0	6	0	0
**EMD638683**	45	14	0	8	4	6	0	0
**GSK650394**	50	15	4	6	2	3	1	1

**Table 7:** The systematic and energetic parameters for SGK1 before and after ZINC00319000 binding.

Complex	RMSD (nm)	RMSF (nm)	R*g* (nm)	SASA (nm^2^)	Kinetic Energy	Enthalpy	Volume (nm^3^)	Density (g/L)
**SGK1**	0.45	0.14	1.88	135.94	157,875	−817,080	642.38	1023.19
**SGK1-ZINC00319000**	0.46	0.14	1.92	139.74	157,820	−817,087	642.23	1023.56

**Table 8:** The percentage of residues participated in the average structure formation of SGK1.

System	Secondary Structure Content (%)							
	Structure *	Coil	β-Sheet	β-Bridge	Bend	Turn	α-helix	Other ^#^
**SGK1**	50	32	13	2	17	10	25	1
**SGK1-ZINC00319000**	52	30	13	2	16	10	27	2

* Structure = α-helix + β-sheet + β-bridge + Turn; ^#^ Other = π-helix + 3_10_-Helix.
